# Titanium particles inhibit bone marrow mesenchymal stem cell osteogenic differentiation through the MAPK signaling pathway

**DOI:** 10.1002/2211-5463.13678

**Published:** 2023-07-30

**Authors:** Shunyi Tong, Sanhua Fang, Kangjie Ying, Weiwei Chen

**Affiliations:** ^1^ Department of Orthopaedic Surgery Lanxi People's Hospital China

**Keywords:** MAPK pathway, mesenchymal stem cell, osteogenic differentiation, Ti particles

## Abstract

Metallic implants have great application in clinical orthopedics. Implants wear out *in vivo* due to long‐term mechanical loading. The formation of wear debris is one of the long‐term complications of prosthesis. In the case of artificial joint replacement in particular, aseptic loosening is the most common reason for secondary revision surgery. Previous studies suggested that wear debris caused aseptic loosening mainly by promoting osteolysis around the prosthesis. In this study, titanium particles, the most commonly used particles in clinical practice, were selected to simulate wear debris and explore the influence of titanium particles on osteogenic differentiation of mesenchymal stem cells. Our results show that titanium particles can significantly inhibit osteogenic differentiation in a dose‐dependent manner. While engaged in preliminary exploration of the underlying mechanisms, we found that titanium particles significantly affect phosphorylation of ERK1/2, a key component of MAPK signaling. This suggests that the MAPK signaling pathway is involved in the inhibition of osteogenic differentiation by titanium particles.

## Graphical abstract

The formation of wear debris is one of the long‐term complications of prosthesis. Wear debris cause aseptic loosening mainly by promoting osteolysis. In this study, we found that titanium particles can significantly inhibit osteogenic differentiation in a dose‐dependent manner through the MAPK signaling pathway.

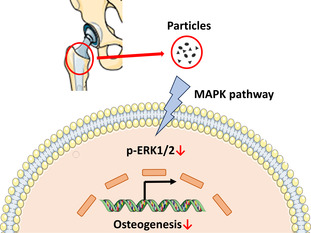

**Abbreviations**
Al: aluminum
ALP: alkaline phosphatase
FBS: fetal bovine serum
GAPDH: glyceraldehyde 3‐phosphate dehydrogenase
rMSCs: rat mesenchymal stem cells
SEM: scanning electron microscope
Si: silicon
Ti: titanium

Orthopedic implants made of metal materials are an important part of clinical orthopedic applications. Compared with traditional medical inorganic nonmetallic materials (bioceramic, bioglass, etc.) and medical polymer materials (polylactic acid, polyglycolic acid, etc.), medical metal materials have better comprehensive mechanical properties and excellent processing and forming ability [1, 2, 3]. The mechanical strength of metal materials is closer to that of human bones, which makes metal materials ideal for the reconstruction of human mechanics. At present, after many years of clinical application of orthopedic metal materials for medical use, there are still many problems, such as autoimmune reaction after material implantation, mismatch between elastic modulus and host bone, nonfusion between bone and interface, as well as the impact of metal corrosion and wear [4, 5].

The production of wear debris is considered to be the key cause of aseptic loosening of orthopedic implants, especially after artificial joint replacement. Due to the fretting and long‐term wear between the implants and host bone tissue, a large number of micron and nanoparticles will accumulate around the implants in the middle and far postoperative period, and the accumulation of wear debris around the implants will change the microenvironment around the endoplant [6]. Specifically, wear debris induce osteoblasts, osteoclasts, dendritic cells, and fibroblasts around the prosthesis to produce a large number of cytokines and chemokines, causing a series of cellular reactions such as chronic inflammation, and destroying the balance of bone remodeling between osteoblasts and osteoclasts, eventually leading to osteolysis and aseptic loosening around the prosthesis [7, 8]. Studies have shown that approximately 55% of total hip replacements and 31% of total knee replacements result from medium‐ and long‐term aseptic loosening of the prosthesis [9]. Therefore, a better understanding of the mechanism of aseptic loosening caused by wear debris will help to reduce the rate of postoperative revision and extend the service life of implants.

Wear debris induced enhanced osteoclast bone resorption activity, accompanied by the release of inflammatory factors, thus causing osteolysis around the prosthesis, which is believed to be the main cause of aseptic loosening of the prosthesis. For example, wear debris could promote osteoclastogenesis through RANK‐activated NF‐κB/MAPK/AKT pathways [10]. Other studies have shown that blockade of NF‐κB and MAPK pathways attenuates wear particle‐stimulated osteoclast differentiation *in vitro* and *in vivo* [11]. In addition, wear debris can stimulate macrophages to release a large number of inflammatory mediators and cytokines (TNF‐α, IL‐6, etc.). These inflammatory mediators and cytokines are activated under the regulation of signaling pathways and act on osteoclast precursors to differentiate and proliferate, transform into mature osteoclasts, and induce osteolysis [12].

On the contrary, wear debris can directly act on osteoblasts, resulting in the change of cell phenotype and the transformation of corresponding genes, and then affect the abnormal expression of proteins in cells, which is ultimately manifested as the weakening of mineralization and proliferation ability of osteoblasts, leading to periprosthesis osteolysis. In addition, the effect of wear debris on osteoblasts can promote the upregulation of the expression of bone resorption factors, such as RANKL, IL‐1β, and PGE2, while the expression of inhibitory bone resorption factors, such as OPG, can promote the overactivation of osteoclasts, thus enhancing the effect of local bone destruction and bone resorption [13, 14]. Therefore, further understanding of the role of osteoblasts in the process of periprosthesis osteolysis will help to find new therapeutic targets for intervention in periprosthesis osteolysis and aseptic loosening.

In this study, titanium, the most widely used metal in clinical application, was selected as the wear particle material to prepare titanium alloy wear debris. Subsequently, we investigated the effect of titanium alloy wear debris on osteogenic differentiation of rat mesenchymal stem cells (rMSCs) *in vitro* and preliminarily explored its possible signaling pathway. Our results suggest that titanium particles can inhibit osteogenic differentiation of rMSCs through MAPK pathway (Fig. 1).

**Fig. 1:**
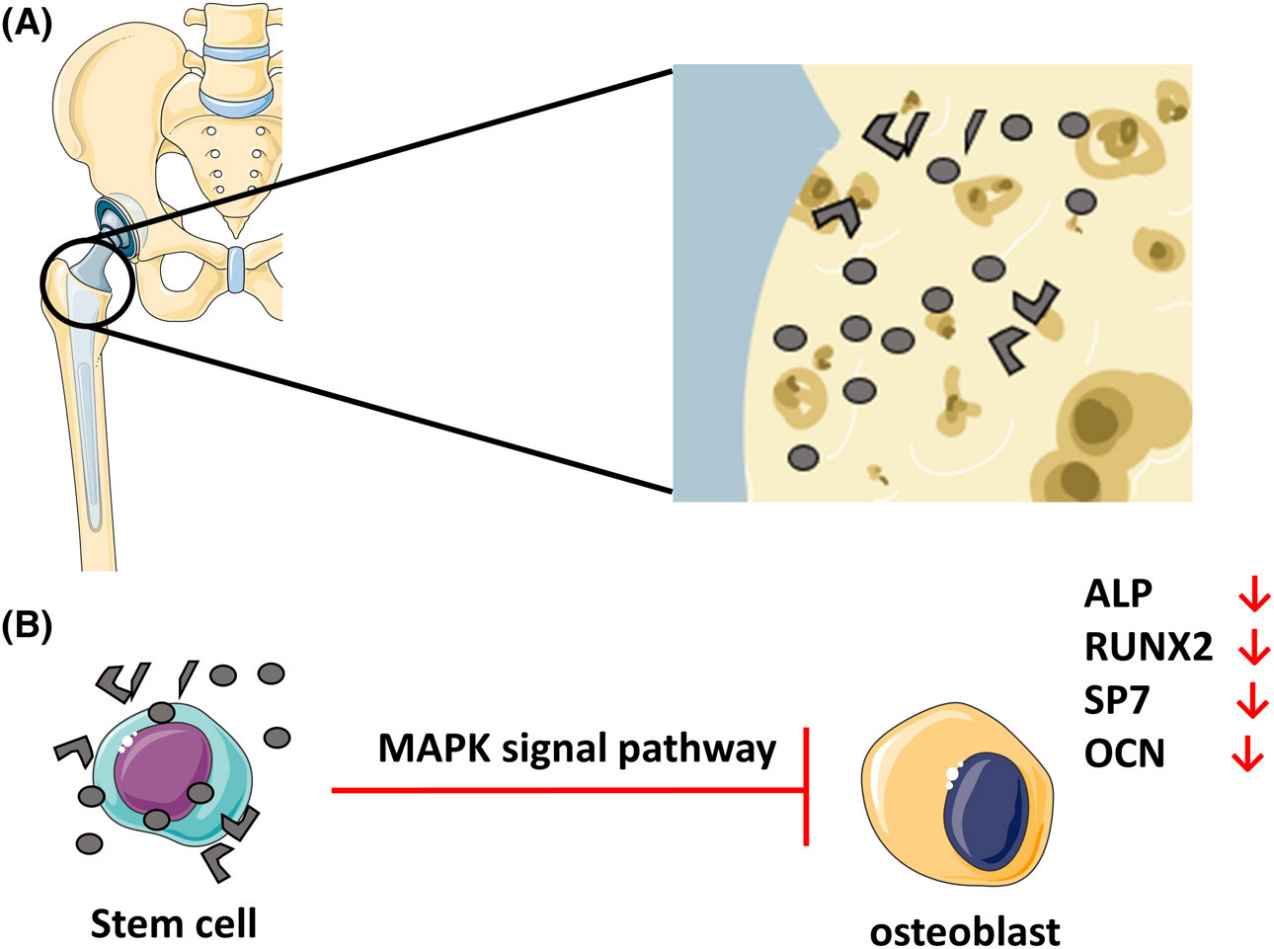
Model diagram of this study. (A) Schematic illustration of particles generated by wear of the prosthesis *in vivo*. (B) Ti particles inhibit osteogenic differentiation through MAPK pathway.

## Materials and methods

### Titanium particles preparation

Titanium (Ti) particles (α + β type titanium alloy, TC4, China) were obtained from XFNAN (Nanjing, China) and were prepared as previously described [15]. Briefly, Ti particles were sterilized by baking at 180 °C for 12 h and then immersed in 75% ethanol for 48 h and rinsed for three times with double‐distilled water at room temperature. Limulus amoebocyte lysate assay (Biowhittaker, Walkersville, MD, USA) was used to detect the endotoxin content of Ti particles. And only particles with 0.02 EU·mL^−1^ endotoxin were used in this study.

### Scanning electron microscopy (SEM)

To evaluate the characterization of Ti particles, they were coated with gold–palladium and observed under a scanning electron microscope (SEM, Hitachi TM‐1010, Tokyo, Japan). Meanwhile, X‐ray energy dispersive analysis was performed to analyze the elemental composition of Ti particles.

### Cell culture and differentiation

Commercial rMSCs (Cyagen Biosciences Inc., Guangzhou, China) were cultured with MSC basal medium containing alpha‐MEM with 1% penicillin and streptomycin supplemented with 10% fetal bovine serum (FBS) in a 37 °C, 5% CO_2_ and 90% humidity incubator. Cells were then cultured in 24‐well plates (4 × 10^4^ cells per well) or 6‐well plates (2 × 10^5^ cells per well) and grown in osteogenic medium containing β‐glycerophosphate, dexamethasone, ascorbic acid, and MSC basal medium. After confluence, the cells were co‐cultured with Ti particles (0, 0.1, 0.2 mg·mL^−1^).

### CCK‐8 assay and live/dead cell staining assay

rMSCs were seeded on 96‐well plates and allowed to attach for 12 h in alpha‐MEM containing 10% FBS. Cells were then cultured with Ti particles (0, 0.1, 0.2 mg·mL^−1^) for 1, 2, 3, 5, and 7 days. At each time point, cells were incubated with fresh serum‐free medium containing the CCK‐8 (Dojindo Lab, Tokyo, Japan) reagent for 2 h at 37 °C. The absorbance was measured using microplate reader at 450 nm.

For live/dead cell staining assay, the cells were plated in 24‐well plates and cultured using the same procedure. After co‐culturing the cells with Ti particles (0, 0.1, 0.2 mg·mL^−1^) for 48 h, the cells were stained with 5 μm Calcein‐AM and 0.6 μm propidium iodide (Life Technologies, Carlsbad, CA, USA) for 30 min. The live/dead cell images were carried out using an inverted microscope (LSM700, Zeiss, Oberkochen, Germany).

### Alkaline phosphatase (ALP) staining

Alkaline phosphatase staining was performed on the cells after 3 or 7 days of culture in osteogenic medium. In simple terms, after being fixed in 4% paraformaldehyde for 10 min, it was rinsed three times with PBS and incubated in BCIP/NBT working solution in the dark for 10 min. The staining results were analyzed under microscope.

### Alkaline phosphatase activity

Alkaline phosphatase activity was determined at 405 nm using p‐nitrophenyl phosphate. Briefly, after 3 days of co‐culture with Ti particles, the cell layers were washed in PBS to remove residual particles and detached cells. Then, the cells were lysed in a solution containing 10 mm Tris and 0.1% Triton X‐100 (pH 7.5). After centrifugation, the supernatants were collected and incubated in an alkaline buffer (pH 10.5) containing 5 mm pNPP and 2 mm MgCl_2_. Then, ALP activity was determined using the methods previously described.

### Quantitative reverse transcriptase polymerase chain reaction

Total RNA was extracted from rMSC cells using TRIzol Reagent (Invitrogen, Carlsbad, CA, USA). RNA integrity was assessed by light absorbance at 260 and 280 nm and by agarose gel electrophoresis with ethidium bromide staining. A housekeeping gene glyceraldehyde 3‐phosphate dehydrogenase (GAPDH) was used as internal control. All reactions were performed in triplicate and analyzed by the $2^{-\Delta \Delta C_{T}}$ method. Primers used: GAPDH forward‐GGCAAGTTCAACGGCACAG, reverse‐CGCCAGTAGACTCCACGACAT; ALP forward‐GTGGTATTGTAGGTGCTGTGGTC, reverse‐CGGTGTCGTAGCCTTCTGG; RUNX2 forward‐CTTCGTCAGCGTCCTATC, reverse‐CTTCCATCAGCGTCAACA; SP7 forward‐GTTCACCTGTCTGCTCTG, reverse‐GGCTGATTGGCTTCTTCT; OCN forward‐GTAAGGTGGTGAATAGACTCC, reverse‐AACGGTGGTGCCATAGAT.

### Western blotting

To precisely evaluate the expression of key factors such as Runx2, ERK1/2, and p‐ERK1/2 in the process of osteogenic differentiation, western blotting was performed. Protein quantification was performed using the BCA protein assay reagent (Beyotime, Shanghai, China). In short, protein samples were collected from cell lysates after incubation with Ti particles (0, 0.1, and 0.2 mg·mL^−1^) for 72 h and then separated with SDS/PAGE, thereafter shifted to a PVDF membrane, which had been blocked and probed with primary antibodies against Runx2 (1 : 1000, Abcam, Cambridge, MA, USA), ERK1/2 (Cell Signaling Technology, Boston, MA, USA) and p‐ERK1/2 (Cell Signaling Technology) overnight at 4 °C. Subsequently, fluorescent‐conjugated secondary antibody diluted in dilution buffer was added to the membranes after washing three times with TBST for 5 min, and the membranes were then incubated at room temperature for 1 h in the dark. Protein quantification was performed using the Odyssey IR software (LI‐COR, Lincoln, NE, USA). As a loading control, anti‐α‐tubulin (Cell Signaling Technology) antibodies were used.

### Statistical analysis

The data are expressed as mean ± standard deviation (SD). The two‐tailed Student's *t*‐test was utilized to make comparisons between groups and one‐way analysis of variance (ANOVA) to perform multiple comparisons. The differences were considered significant when *P* < 0.05. (*) for *P* < 0.05, (**) for *P* < 0.01, and (***) for *P* < 0.001.

## Results

### Characterization of Ti particles

**Fig. 2:**
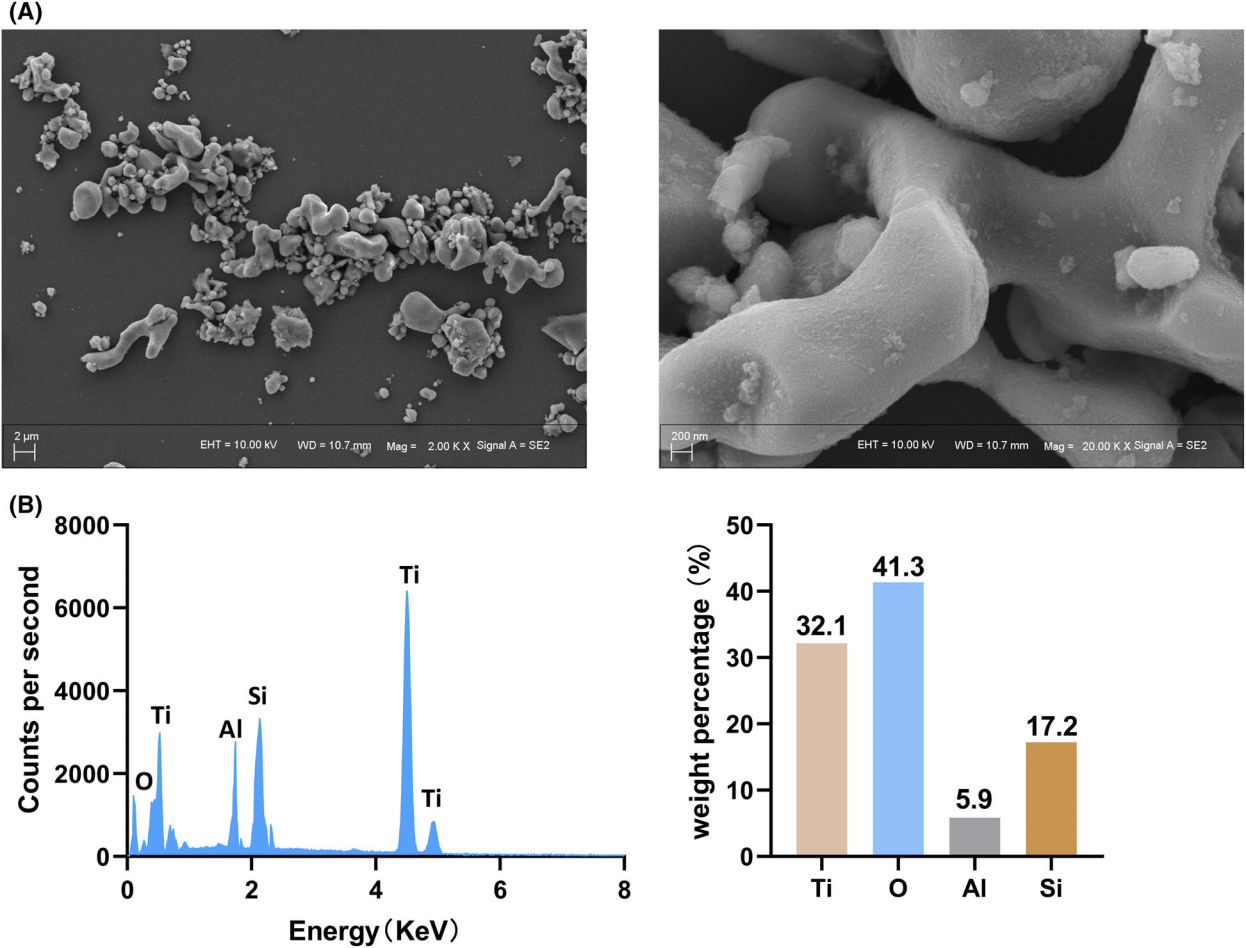
Characterization of Ti particles. (A) SEM images of Ti particles. Scale bar: 2 μm (left); 200 nm (right). (B) The elemental composition of Ti particles.

First, SEM was used to gain an insight into the microstructure of Ti particles. Ti particles are in the micron scale and have different sizes and irregular shapes (Fig. 2A). From the picture, we can see that the smallest Ti particles are < 1 μm in diameter, while the largest Ti particles are about 4 microns in diameter. Moreover, in the magnification we can see that the Ti particles are uneven rather than smooth. This may more closely resemble the disuniform, irregular wear debris of prostheses *in vivo*. In addition, we also analyzed the elemental composition of Ti particles (Fig. 2B). The results show that titanium and oxygen are the main components of Ti particles, and other elements include silicon (Si) and a small amount of aluminum (Al).

### Biocompatibility of Ti particles

**Fig. 3:**
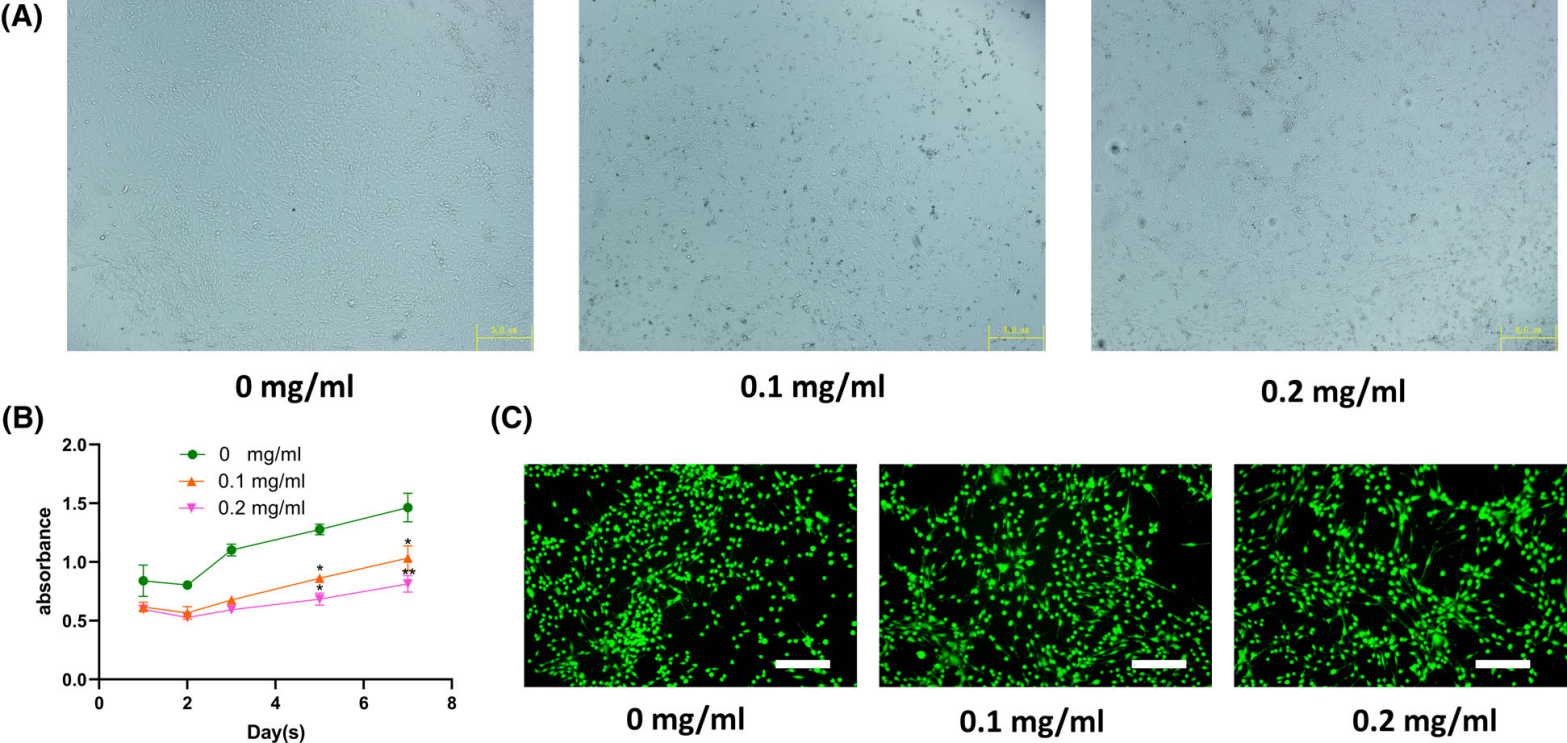
Biocompatibility of Ti particles. (A) Microscopic image of Ti particles and cell co‐culture. Scale bar: 5 μm. (B) Viability of rat mesenchymal stem cells (rMSCs) quantified by CCK‐8 assay. (C) Live/dead staining of rMSCs cultured with Ti particles after 48‐h incubation. Live cells were stained as green and dead cells were stained as red. Scale bar: 200 μm. Data are presented as mean values ± standard deviation; *n* = 3; Student's *t*‐tests (*P < 0.05, **P < 0.01).

Before testing the biocompatibility of Ti particles, we first observed the state of cells in co‐culture with Ti particles through a microscope (Fig. 3A). This is mainly to observe the position of Ti particles when they are co‐cultured with cells. It turns out that the Ti particles stick to the surface of the cells or enter the cells because they stay put when we shake the plate. Next, the live/dead assay and CCK‐8 assay were used to detect the effect of Ti particles on cell proliferation and survival. Ti particles had no significant effect on cell proliferation and survival in first 3 days (Fig. 3B,C). However, with the extension of culture time, the toxic effect of Ti particles on cells gradually appeared.

### Ti particles inhibit osteogenic differentiation of rMSC cells

**Fig. 4:**
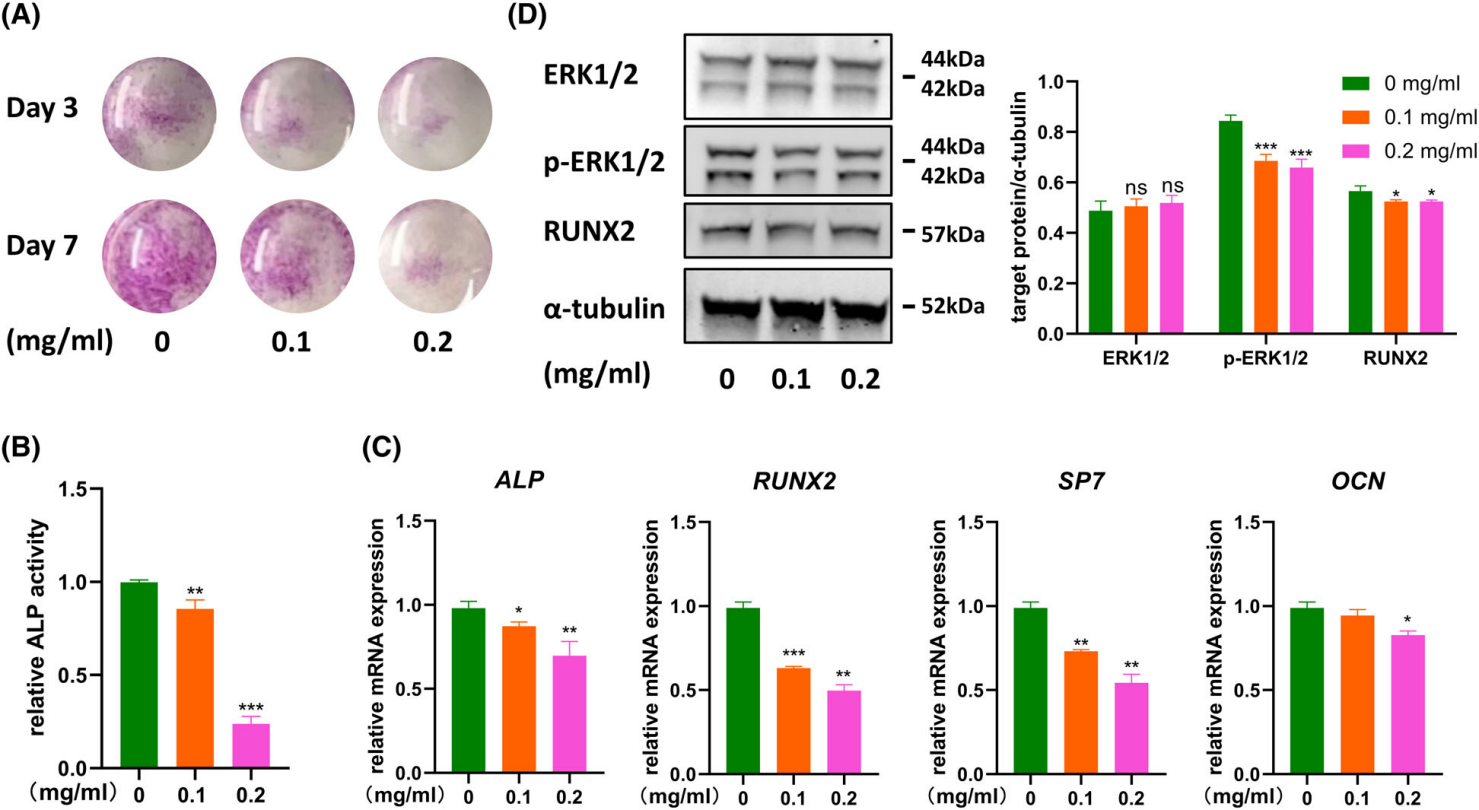
Ti particles inhibit osteogenic differentiation of rat mesenchymal stem cells (rMSC). (A, B) Alkaline phosphatase (ALP) staining and ALP activity analysis were performed to evaluate the expression of ALP in rMSCs after co‐culture with Ti particles for 3 and 7 days. (C) Osteogenic marker genes were detected to assess the level of cell differentiation by qPCR. (D) Runx2, ERK1/2, and p‐ERK1/2 were determined by western blot. Data are presented as mean values ± standard deviation; *n* = 3; Student's *t*‐tests (*P < 0.05, **P < 0.01, ***P < 0.001).

Next, we examined the effect of Ti particles on osteogenic differentiation of rMSCs. First, we performed ALP staining and activity detection. Ti particles significantly inhibited osteogenic differentiation in a dose‐dependent manner (Fig. 4A,B). It was consistent with the fact that the marker genes of osteogenesis were also significantly reduced under the treatment of Ti particles (Fig. 4C). Finally, at the protein level, we also confirmed the decline of RUNX2 protein. What's interesting about this is that when it comes to detecting ERK1/2, a key protein in the MAPK signaling pathway. We found no significant changes in total ERK1/2 protein, but significant reductions in functionally phosphorylated ERK1/2 protein (Fig. 4D). This suggests that Ti particles may inhibit osteogenic differentiation of rMSCs through MAPK signaling pathway.

## Discussion

Poor integration of metallic implants and host bone is the key cause of aseptic loosening [6, 16]. Osteolysis induced by wear debris is thought to be the key to the initiation of aseptic loosening. Current studies have shown that wear debris stimulate macrophages, osteoclasts, lymphocytes, and so on, causing a series of biological changes in aseptic inflammatory response and activating osteoclasts [17, 18].

At present, the main biological cause of aseptic loosening is the osteoclastic bone resorption induced by macrophage mediated by wear particles. At the same time, inflammatory responses are closely related to overactive bone resorption. The primary condition of aseptic loosening is the release of inflammatory factors. The inflammatory mediators such as IL‐1 and IL‐6 produced by the wear particles acting on the tissue around the prosthesis can induce the formation and activation of osteoclasts, and eventually lead to osteolysis. Therefore, inhibiting inflammation or inhibiting osteoclast activation and differentiation can effectively prevent the occurrence and development of aseptic loosening. For example, studies have shown that biochanin A downregulated the secretion levels of TNF‐α, IL‐1α, and IL‐1β to suppress inflammatory responses. At the same time, it can inhibit the activation of osteoclasts induced by wear particles through NF‐κB and MAPK pathways [19]. And another study has shown that the V‐ATPase inhibitors also impaired osteoclast differentiation via the inhibition of RANKL‐induced NF‐κB and ERK signaling pathways, which are potential antiresorptive agents for the treatment of peri‐implant osteolysis [20].

However, wear debris not only affect bone homeostasis directly by stimulating osteoclast generation but also adversely affect osteoblast generation. Osteoblasts are derived from pluripotent mesenchymal stem cells in the inner and outer periosteum and in the bone marrow stroma. Osteoblasts are mainly composed of immature osteoblast precursor cells, osteoblasts in the process of differentiation, and mature osteoblasts with the ability of bone stroma formation. The host response of osteoblasts to wear debris is critical for bone formation around the prosthesis. More and more studies have demonstrated that wear debris impairs the function of mature osteoblasts and inhibits bone formation and differentiation of osteoblast precursors [21, 22, 23], which is consistent with our results. However, the mechanism by which wear debris inhibit bone formation remains to be elucidated.

Although osteoblasts are nonphagocytic cells, they can still transfer wear debris into the cytoplasm through cytophagocytosis or micropinocytosis and through endocytosis mediated by clastun and concavin. The transfer of wear debris into the cell alters the cell differentiation. It has been reported that pretreatment with cytophagocytosis inhibitor Cytochalasin D can inhibit endocytosis of cells, thereby reducing the activity, differentiation and inflammatory response of cells induced by wear debris [24]. Wear debris can directly damage organelles, cause DNA damage, activate DNA repair, destroy the structure and function of cytoskeleton, and affect the viability, proliferation, adhesion, migration, and other cell activities of osteoblasts [25, 26]. Moreover, the differentiation and mineralization ability of osteoblasts are also decreased under the influence of wear debris. It was also observed in our results that wear debris were not suspended in the culture medium, but were anchored to the cells. At the same time, our study showed that Ti particles can inhibit the differentiation of rMSCs into osteoblasts. The expressions of ALP, RUNX2, SP7, and other osteogenic genes decreased after co‐culture of rMSCs with wear debris.

In addition, studies have shown that wear debris can also indirectly promote bone resorption by acting on osteoblasts. When wear debris activated osteoblasts and promoted the secretion of inflammatory cytokines, the secretion of pro‐inflammatory cytokines (including TNF‐α, IL‐1, and IL‐6) and the expression of related genes increased significantly in a time‐ and concentration‐dependent manner [27, 28]. These pro‐inflammatory factors further activate osteoclasts and promote osteoclast bone destruction, ultimately leading to the formation of osteolysis around the prosthesis. Wear debris can also recruit inflammatory cells to activate the inflammatory response around the prosthesis, which plays a key role in osteolysis around the prosthesis. For example, mRNA expression and protein secretion of MCP 1 and IL‐8 in osteoblasts were significantly increased, and these promoted the progression of inflammation [29].

A variety of signaling pathways including NF‐κB, RANKL, and Wnt/β‐catenin are involved in the induction of periprostheses osteolysis by wear debris stimulating osteoblasts. The Wnt/β‐catenin signaling pathway, which is regulated by ubiquitin‐mediated proteasomal degradation of β‐catenin, plays a key role in osteoblast differentiation and bone formation. Studies have shown that GSK‐3β inhibitor AR28 modulates the Wnt/β‐catenin and BMP 2 signaling pathways to upregulate osteoblast differentiation, accelerate osteoblast repair, and reduce osteoclast generation in rat models of periprosthesis osteolysis [30]. Other studies have shown that wear debris can inhibit GSK‐3β phosphorylation, further inhibit osteoblast differentiation through GSK‐3β/β‐catenin pathway, and exacerbate osteolysis [31]. Osteoblasts can also influence periprosthogenic osteolysis through receptor activator‐dependent signaling pathways of NF‐κB receptor activator ligands. The activator of NF‐κB receptor activator ligand activates osteoclasts by binding to the NF‐κB receptor activator ligand receptor on the surface of the cell membrane of osteoclasts' precursors, triggering a cascade of NF‐κB signaling pathways that are essential for osteoclast differentiation [32]. OPG secreted by osteoblasts is an osteoclast suppressor that inhibits the receptor activator activity of NF‐κB receptor activator ligand. RNAKL/OPG ratio is a critical regulator for osteoclast activation. The study showed that the RANKL/OPG ratio increased significantly in osteoblasts and surrounding tissue cells induced by various wear debris [33, 34].

In this study, we preliminarily explored the role of MAPK signaling pathway in the inhibition of osteogenic differentiation by titanium particles. Our results suggest that titanium particle treatment can affect ERK1/2 phosphorylation, thereby altering cell function. MAPK signaling pathway plays an important role in bone development and bone metabolism. For example, naringin has been reported to promote osteogenic differentiation of stem cells while affecting the activation of ERK signaling [35]. Mechanical stress can enhance osteogenic differentiation of stem cells through MAPK signaling pathway [36, 37].

In addition, MAPK signaling pathway may also play a synergistic role with NF‐κB signaling pathway. A number of studies have shown that titanium particle‐induced osteolysis can be alleviated through NF‐κB and MAPK pathways [11, 38, 39]. However, NF‐κB and MAPK pathways not only play a role in titanium particle‐induced osteoclasts but also other cells can respond to titanium particle‐induced stimulation through these pathways. For example, MG‐63 cells exposed to titanium particles not only promote binding of the transcription factor NF‐κB to the IL‐8 promoter but also activate the MAPK signaling pathway [40]. MAPK and NF‐κB signaling pathways promote chronic inflammation and downregulate collagen synthesis in osteoblasts by upregulating the production of inflammatory cytokines and chemokines. MAPK signaling pathway can regulate the activity of osteoblasts under the induction of MMP 2. MC3T3E‐1 cells were co‐cultured with titanium particles to activate MAPK signaling pathway and trigger the expression of MMP 2. However, the p38 inhibitor SB203580 inhibited the mRNA expression and protein secretion of MMP 2 induced by titanium particles [41]. It can be seen that titanium particles are very likely to play a role through MAPK signaling pathway, which is preliminarily confirmed by our results.

However, our results are only a preliminary exploration and further research is still needed. In terms of mechanism, the relationship between ERK1/2 signal expression and endocytosis of titanium particles needs to be explored. Based on previous reports, MAPK signaling pathway is involved in cellular endocytosis [42, 43]. Therefore, it is reasonable to speculate that the MAPK signaling pathway is activated during the uptake of titanium particles into cells. In addition, we need to construct the implant model later and inject different concentrations of titanium particles around the implant to explore the effect of titanium particles *in vivo*. At the same time, we can use drugs to inhibit the activation of MAPK signaling pathway, or cytochalasin to inhibit the endocytosis of cells, so as to explore the specific mechanism of the role of titanium particles.

In conclusion, our results preliminarily confirmed that titanium particles not only act on mononuclear macrophages or osteoclasts but also act on mesenchymal stem cells through MAPK pathway, providing new insights for aseptic loosening around prosthesis. And the follow‐up study is expected to provide a new therapeutic strategy for clinical treatment.

## Conclusions

In this study, we chose titanium particles to simulate the prosthesis wear debris *in vivo*. Ti particles have the characteristics of uneven size and irregular shape. First, we tested the characterization and biocompatibility of Ti particles. Second, we examined the effect of Ti particles on osteoblast differentiation of rMSCs. Our results showed that Ti particles significantly inhibited osteogenic differentiation in a dose‐dependent manner. Finally, the protein detection results suggested that Ti particles might inhibit osteogenic differentiation through MAPK signaling pathway.

## Conflict of interest

The authors declare no conflict of interest.

### Peer review

The peer review history for this article is available at https://www.webofscience.com/api/gateway/wos/peer‐review/10.1002/2211‐5463.13678.

## Author contributions

The experiments were designed by ST. The experiments were performed by SF and KY. WC collected and analyzed data. SF wrote and edited the manuscript. ST revised the manuscript. All authors read it and finally approved the final version.

## Data Availability

Data will be made available on request.
